# Cutaneous *Naganishia albida* (*Cryptococcus albidus*) infection: a case report and literature review

**DOI:** 10.1590/S1678-9946202365060

**Published:** 2023-12-04

**Authors:** Vítor Falcão de Oliveira, Alexandre Pereira Funari, Mariane Taborda, Adriana Satie Gonçalves Kono Magri, Anna Sara Levin, Marcello Mihailenko Chaves Magri

**Affiliations:** 1 Universidade de São Paulo, Faculdade de Medicina, Hospital das Clínicas, Divisão de Infectologia, São Paulo, São Paulo, Brazil

**Keywords:** Cryptococcus albidus, Naganishia albida, Cryptococcal infection, Fungal skin disease

## Abstract

*Naganishia albida* (*Cryptococcus albidus*) is considered saprophytic fungi, and is rarely reported as a human pathogen. Cutaneous infections caused by non-neoformans cryptococcus are rare. We describe a case of an immunocompetent older male with cutaneous cryptococcosis caused by *Naganishia albida* following skin trauma, and conduct a literature review in PubMed, Lilacs, and Embase. Only six previous similar reports were found. The seven cases (including ours) were widely distributed geographically (Brazil, the US, the UK, Hungary, South Korea, and Iran), all males, and their ages varied, ranging from 14 to 86 years. Four individuals had underlying skin diseases (Sezary Syndrome, psoriasis, and skin rash without etiology) plus potentially immunosuppressive underlying conditions (diabetes mellitus, kidney transplantation, and the use of etanercept, adalimumab, and methylprednisolone). Cutaneous presentation was polymorphic, with lesions characterized as warts, ulcers, plaques, and even macules. Two patients presented disseminated disease. Serum cryptococcal antigen was negative in six patients, and diagnosis was made by fungal culture in all. There is a lack of data on optimal antifungal treatment and outcomes.

## INTRODUCTION

Cryptococcosis is an opportunistic yeast infection, and *Cryptococcus neoformans* is the most prevalent human pathogenic species complex^1^. Skin involvement due to *C. neoformans* are found in almost 5% of patients with cryptococcal meningitis^2^. Skin lesions are generally attributable to hematogenous dissemination, and the association with a skin portal of entry is still controversial^2^.

Non-neoformans species, such as *Cryptococcus albidus*, which was reclassified as *Naganishia albida* ^3^, have been identified from various environmental sources. They are considered saprophytic fungi, and are rarely reported as human pathogens^4^. Infections caused by non-neoformans cryptococci are rare^5^, but their incidence has increased^4^. Data in the literature on this condition is scarce, especially isolated skin infections.

Here we describe a case of cutaneous cryptococcosis caused by *Naganishia albida*, and perform a literature review for other cases, to describe demographic and clinical characteristics, antifungal treatment, and outcomes of this rare disease. To our knowledge, only six cases with cutaneous lesions due to *Naganishia albida* were published.

## CASE REPORT

**Figure 1:**
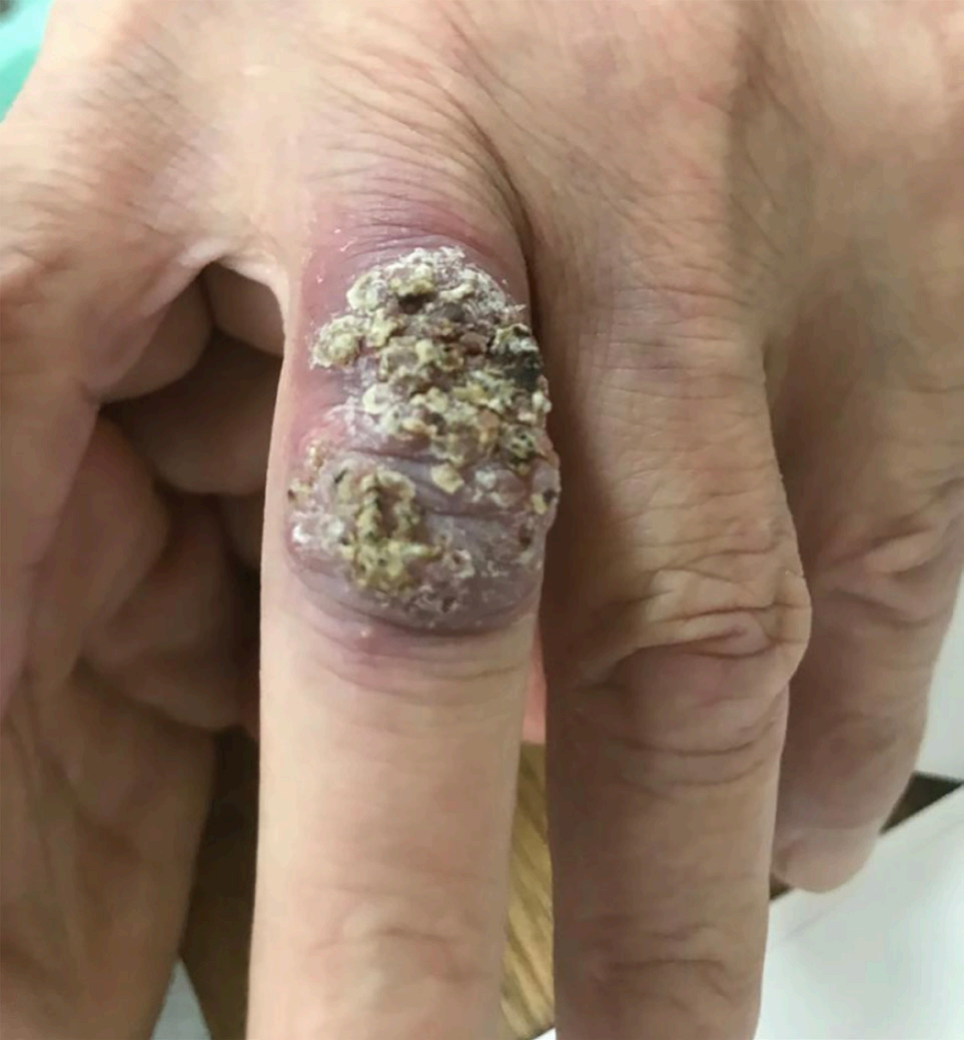
Cutaneous cryptococcosis due to *Cryptococcus albidus* at the diagnosis, before starting the antifungal therapy.

**Figure 2:**
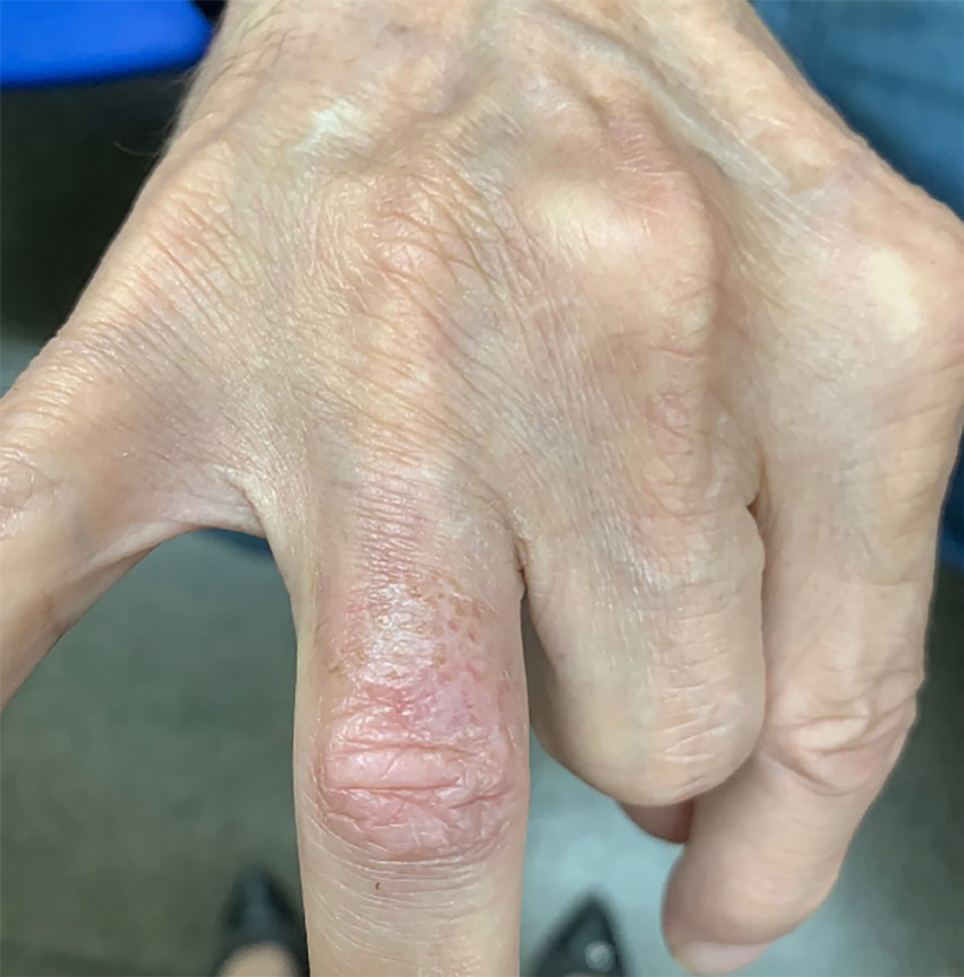
The lesion cleared after 7 months of oral fluconazole.

A 72-year-old Brazilian retired engineer, with hypertension, presented to our medical center with a 10-month history of a lesion on the third finger of his left hand. The lesion had 3 centimeters, with a warty aspect, and without any secretion or bleeding (Figure 1). The base was discreetly erythematous. It had started as a small cut after manipulating an aquarium and the lesion had grown progressively, causing no pain, pruritus, or systemic symptoms. He had received potassium iodide for 45 days, and itraconazole for 5 months, as empirical treatment for sporotrichosis, with no clinical response. The patient was then referred to our outpatient clinic. We performed a skin biopsy. Histological examination showed intense hyperparakeratosis, and pseudoepitheliomatous hyperplasia. In the dermis, a dense lymphohistiocytic infiltrate was observed with epithelioid histiocytes and multinucleated giant cells, with few neutrophils, and vascular tissue proliferation with red blood cell extravasation extending to the hypodermis. Direct microscopy using Gram and KOH staining were negative, but *Naganishia albida* grew in a fungal culture with brain-heart-infusion broth. Positive sample was identified through the automated VITEK2 system (bioMérieux, Marcy-l’Étoile, France) and matrix-assisted laser desorption/ionization time-of-flight mass spectrometry (Bruker Daltonics, Bremen, Germany). Acid-fast stains and culture tests were negative for mycobacteria. Antifungal susceptibility testing was not performed. Serum cryptococcal antigen by latex agglutination resulted negative. Chest computed tomography was normal, and blood laboratory tests were unremarkable. Then, 300 mg fluconazole per day was started, and we reduced the dose to 150 mg per day after 4 months of treatment. Cutaneous infection completely resolved after 7 months of oral fluconazole (Figure 2).

## RESULTS AND DISCUSSION

In our case of cryptococcosis by *Naganishia albida* in an immunocompetent patient the skin was the only organ affected. It is probable that the patient was inoculated directly through a traumatic skin injury. Due to the rarity of this condition, we decided to review the literature to characterize the disease.

We searched PubMed, Lilacs, and Embase for a literature review on September 10, 2023. Our search terms were “*Cryptococcus albidus*”, “*Naganishia albida*” and “Cutaneous infection”, without any restrictions regarding language or publication date. We found only six case reports^5–10^. Including our case, we totaled seven patients with cutaneous infection due to *Naganishia albida* (*Cryptococcus albidus*), summarized in Table 1.

**Table 1:** Demographic and clinical characteristics, antifungal treatment, and outcomes of 7 patients with cutaneous cryptococcosis due to *Cryptococcus albidus*

Article	Year	Country	Age	Sex	Underlying skin disease	Characteristic of the cutaneous lesions	Potentially immunosuppressive conditions	Disseminated disease	Diagnosis	Serum cryptococcal antigen	Treatment	Duration of treatment	Outcome
This case report	2023	Brazil	72	Male	No	Warts	No	No	Fungal culture	Negative	Fluconazole	7 months	Cutaneous infection completely resolved
Gharehbolagh *et al*.^8^	2017	Iran	26	Male	No	Hyperpigmented patch	No	No	Direct microscopic examination and fungal culture	NA	Itraconazole	NA	Cutaneous infection completely resolved
Gyimesi *et al*.^5^	2017	Hungary	86	Male	Skin rash, without etiology	Ulcerated lesions	Yes; Methylprednisolone	Yes	Fungal culture, and histopathology	Negative	Fluconazole	5 months and 2 weeks	Cutaneous infection completely resolved
Endo *et al*.^9^	2011	US	83	Male	Psoriasis	Hemorrhagic crusted plaque	Yes; Use of Etanercept, adalimumab, efalizumab, and tacrolimus ointment	No	Fungal culture	Negative	Fluconazole	6 months	Cutaneous infection completely resolved
Hoang *et al*.^6^	2007	US	14	Male	Psoriasis	Plaque with purulent material.	Yes; Use of Etanercept	No	Fungal culture	Negative	Fluconazole	NA	Cutaneous infection completely resolved
Lee *et al*.^10^, Belda *et al.* ^11^	2004	South Korea	23	Male	No	Erythematous macules and patches	Yes; Kidney transplant, use of cyclosporine and prednisone	Yes	Fungal culture, and histopathology	Positive	Fluconazole	12 months	No evidence of recurrent cryptococcal infection
Narayan *et al*.^7^	2000	UK	70	Male	Sézary syndrome	Ulcerated lesions	Yes; *Diabetes mellitus*	No	Fungal culture, and histopathology	Negative	Fluconazole	2 months	NA

NA = Information not available

Non-neoformans species are widely distributed geographically^4^, and we observed cases described in the Americas, Europe, and Asia. All patients were male and ages ranged widely, from 14 to 86 years. A study showed that patients with primary cutaneous cryptococcosis were older than patients who had secondary cutaneous cryptococcosis or other forms of cryptococcosis^2^. Most patients had an immunosuppressive condition associated with an underlying skin disease. A smaller percentage of patients had underlying immunosuppression with primary cutaneous cryptococcosis^2^. Cutaneous *Naganishia albida* infection were predominantly primary, only two cases had disseminated disease with lung involvement.

Generally, cutaneous cryptococcal infections are polymorphic, hindering diagnosis^11^. This also was observed in our study with *Naganishia albida*, with lesions characterized as warts, ulcers, plaques, and even macules. The cryptococcal antigen tests are commonly negative^7^. Almost all patients in our study had a negative cryptococcal antigen test. For this reason, the diagnosis depended on fungal culture, which was positive in all cases. Notably, histopathology and direct examination are also techniques that make diagnosis more robust.

## CONCLUSION

In conclusion, cutaneous cryptococcosis by *Naganishia albida* is an extremely rare condition, widely distributed geographically. It seems to have an extremely variable presentation, and does not seem to affect a specific age group, however all patients were male. Underlying diseases were present in four patients but the other two had no known health conditions. As lesions were diverse, the diagnosis was difficult and depended basically on culture. There is still a lack of data on optimal antifungal treatment and outcomes in the literature.

## References

[1] Maziarz EK, Perfect JR (2016). Cryptococcosis. Infect Dis Clin North Am.

[2] Neuville S, Dromer F, Morin O, Dupont B, Ronin O, Lortholary O (2003). Primary cutaneous cryptococcosis: a distinct clinical
entity. Clin Infect Dis.

[3] Liu XZ, Wang QM, Göker M, Groenewald M, Kachalkin AV, Lumbsch HT (2015). Towards an integrated phylogenetic classification of the
Tremellomycetes. Stud Mycol.

[4] Khawcharoenporn T, Apisarnthanarak A, Mundy LM (2007). Non-neoformans cryptococcal infections: a systematic
review. Infection.

[5] Gyimesi A, Bátor A, Görög P, Telegdy E, Szepes E, Kappéter A (2017). Cutaneous Cryptococcus albidus infection. Int J Dermatol.

[6] Hoang JK, Burruss J (2007). Localized cutaneous Cryptococcus albidus infection in a
14-year-old boy on etanercept therapy. Pediatr Dermatol.

[7] Narayan S, Batta K, Colloby P, Tan CY (2000). Cutaneous cryptococcus infection due to C. albidus associated
with Sézary syndrome. Br J Dermatol.

[8] Aghaei Gharehbolagh S, Nasimi M, Agha Kuchak Afshari S, Ghasemi Z, Rezaie S (2017). First case of superficial infection due to Naganishia albida
(formerly Cryptococcus albidus) in Iran: a review of the
literature. Curr Med Mycol.

[9] Endo JO, Klein SZ, Pirozzi M, Pirozzi C, Hull CM (2011). Generalized Cryptococcus albidus in an immunosuppressed patient
with palmopustular psoriasis. Cutis.

[10] Lee YA, Hee JK, Tae WL, Myung JK, Mu HL, Ju HL (2004). First report of Cryptococcus albidus-induced disseminated
cryptococcosis in a renal transplant recipient. Korean J Inter Med.

[11] Belda W, Casolato AT, Luppi JB, Passero LF, Criado PR (2022). Primary cutaneous cryptococcosis caused by Cryptococcus gatti in
an elderly patient. Trop Med Infect Dis.

